# The Phosphate of Zinc

**Published:** 1873-10

**Authors:** 


### THE PHOSPHATE OF ZINC.

Dr. Routh, of London, says :—

A very elegant preparation of phosphorus is the phosphide of zinc. My experience with this medicine is very extensive. I have never known it to produce the least unpleasant effect, and have rarely been disappointed in obtaining the full results to be expected from phosphorus in corresponding doses.

The chemical formula of phosphide of zinc is $6Zn_3$, and consequently one grain represents a little more than 1-7th of a grain of phosphorus. The proper dose is, therefore, 1-7th of a grain. I usually prescribe it in cerebral congestion according to the following prescription:

℞. Zinci phosphidi, gr. iij. ;
Ros. conserv. q. s. ut fiant, pill xxx.
Dose, 1, *ter die.*

Instead of the conserve of roses, gr. x. of ext. nucis vomicæ may be substituted if strychnia is required.—*Med. & Sur. Rep.*

